# Long-Term Survival in Brown-Vialetto-Van Laere Syndrome: A Case Report Highlighting Respiratory Care

**DOI:** 10.7759/cureus.84491

**Published:** 2025-05-20

**Authors:** Joana Lourenço, Sandra Moreira, Miguel Leão, Paula Simão, Luisa Ramos

**Affiliations:** 1 Department of Pulmonology, Pedro Hispano Hospital, Matosinhos Local Health Unit, Matosinhos, PRT; 2 Department of Neurology, Pedro Hispano Hospital, Matosinhos Local Health Unit, Matosinhos, PRT; 3 Neurogenetics Unit, Department of Medical Genetics, São João University Hospital, São João Local Health Unit, Porto, PRT

**Keywords:** airway clearance techniques, brown-vialetto-van laere syndrome, respiratory failure, riboflavin transport deficiency, ventilatory support

## Abstract

Brown-Vialetto-Van Laere syndrome (BVVLS) is an extremely rare genetic neurological disorder caused by riboflavin transport deficiency, an autosomal recessive condition mostly associated with mutations in the *SLC52A2* and *SLC52A3* genes. It follows a progressive course, typically characterized by sensorineural deafness, facial weakness, ponto-bulbar palsy, ataxia, and peripheral sensory-motor neuropathy. This disease is often associated with childhood mortality if left untreated. We report the case of a 68-year-old woman who first noticed a mild hearing loss at the age of 12. This was followed by a slowly progressive onset of bilateral facial paresis, dysarthro-dysphonia, stridor, and tongue atrophy with fasciculations. At 63 years of age, genetic testing revealed a single heterozygous variant in the *SLC52A3* gene. Although typically autosomal recessive, some individuals with classic symptoms and even response to riboflavin therapy have been found to carry only a single mutation in either the *SLC52A2* or *SLC52A3* gene. Therefore, given the compatible clinical presentation, a diagnosis of BVVLS was considered after discussion with a center of expertise. Consequently, 10 mg/kg/day of riboflavin supplementation was prescribed for three years, but no significant clinical improvement was observed. Currently, at age 68, the patient is on nocturnal non-invasive mechanical ventilation (NIV) and uses assisted airway clearance techniques, including air-stacking maneuvers and mechanical insufflation-exsufflation on demand, due to respiratory compromise secondary to diaphragmatic weakness and vocal cord paralysis. This unique presentation of slowly progressive symptoms and long survival may be related to the single heterozygous *SLC52A3* variant found. Respiratory care in BVVLS is currently adapted from other neuromuscular disorders with stronger evidence bases. This case highlights the critical role of pulmonology in BVVLS care, including clinical and functional monitoring, early initiation of NIV, and the implementation of airway clearance techniques.

## Introduction

Riboflavin, also known as vitamin B2, is a water-soluble vitamin acquired exclusively through dietary intake [1]. It serves as a precursor to several coenzymes, essential for carbohydrate, amino-acid, and lipid metabolism [2]. Three riboflavin transporters have been distinguished: RFVT1 and RFVT3 are mostly expressed in the small intestine; RFVT2 is predominantly expressed in nervous tissue [2,3]. 

Riboflavin transporter deficiency (RTD) is a rare neurological condition that refers to biallelic mutations essentially in the *SLC52A2 *or *SLC52A3* genes, which encode RFVT2 and RFVT3, respectively [4]. Although *SLC52A1* mutations (RFVT1 deficiency) can occur, this represents an ultrarare and even poorly understood entity, as only seven cases have been reported, exhibiting different clinical presentations [5-9]. RTD is classically associated with autosomal recessive inheritance, in which the condition manifests in individuals carrying two pathogenic alleles, either as homozygotes (with two copies of the same mutant allele) or as compound heterozygotes (with two different pathogenic variants in the same gene) [10]. However, some patients have been reported with classic RTD features and even dramatic responses to riboflavin therapy, despite not fitting the expected autosomal recessive inheritance pattern [11]. These include individuals with mutations in both *SLC52A2* and *SLC52A3* simultaneously, as well as others carrying only a single pathogenic variant in one of these genes [11].

RTD fundamentally includes Brown-Vialetto-Van Laere syndrome (BVVLS) and Fazio-Londe syndrome (FLS), phenotypically continuous conditions characterized by progressive sensorimotor and cranial neuropathy [3,12]. BVVLS presents with a variable, but often rapidly progressive, sensorineural deafness, ponto-bulbar palsy, and respiratory compromise, while FLS lacks hearing loss [12]. Only 110 RTD cases were reported by 2019, with BVVLS prevalence estimated at less than 1/1,000,000 [4]. It affects specifically female individuals, with symptom onset typically from childhood to early adulthood [3,4,13]. BVVLS frequently begins with progressive severe sensorineural deafness and evolves to include bulbar palsy (dysphagia and dysarthria), facial weakness, optic nerve atrophy, ataxia, muscle hypotonia, and ultimately respiratory involvement [2,3,12]. Definitive diagnosis relies on mutational analysis of riboflavin transporter genes [4]. Untreated, BVVLS can be rapidly fatal at a young age [14]. High-dose riboflavin supplementation is currently the only lifesaving therapy in RTD [3,12]. However, response and prognosis are highly variable, with respiratory failure remaining the leading cause of death [13,14].

This report aims to describe an atypical presentation of BVVLS, marked by late-onset symptoms and a slowly progressive course, with a presumptive diagnosis supported by the presence of a single variant in the *SLC52A3 *gene.

This case was previously presented as a meeting poster at the II Respiratory Care Congress on May 9, 2024, in Oporto, Portugal.

## Case presentation

The patient is a 68-year-old woman with no relevant family history, including five siblings with no neurological symptoms. Her birth and early development were normal. At 12 years of age, mild hearing loss was first noticed. Over the years, she experienced progressive sensorineural deafness and bilateral facial weakness. Medical records mentioned bilateral paresis of VII, IX, X, and XII cranial nerves, but the initial work-up study was inconclusive. Symptoms remained relatively stable for several years.

As a 62-year-old, she suffered profound deafness, requiring lip reading to communicate. Physical examination revealed bilateral facial palsy and fasciculations (worse on the right side), atrophic tongue with constant bilateral fasciculations, dysphonia, and dysphagia. She also presented with brisk deep tendon reflexes in all four limbs but no evident paresis. No sensory or cerebellar abnormalities were present at neurological examination. Electromyography showed chronic cranial motor neuronopathy, affecting facial and bulbar muscles, and no limb neuropathy was found. Cranial MRI and serum analysis were normal, including viral serologies, acylcarnitine profile, and riboflavin levels (243 µg/L; normal 130-300 μg/L). Given the clinical suspicion of BVVLS, sequencing of the *SLC52A2* and *SLC52A3* genes was requested, revealing a single heterozygous variant in *SLC52A3* (c.1238T>C; p.Val413Ala), a missense mutation located in exon 5. This was subsequently confirmed through mendelioma analysis (clinical exome sequencing), with no other relevant findings to report. Additionally, no deletions/duplications of the *SLC52A3* gene were detected. Although this variant was classified as of uncertain significance, it has previously been reported in cases of BVVLS [15-17]. Therefore, following discussion with a center of expertise, the diagnosis of BVVLS presenting with a mild phenotype was considered, despite the identification of only a single heterozygous variant. Genetic testing of the parents could not be conducted.

**Figure 1:**
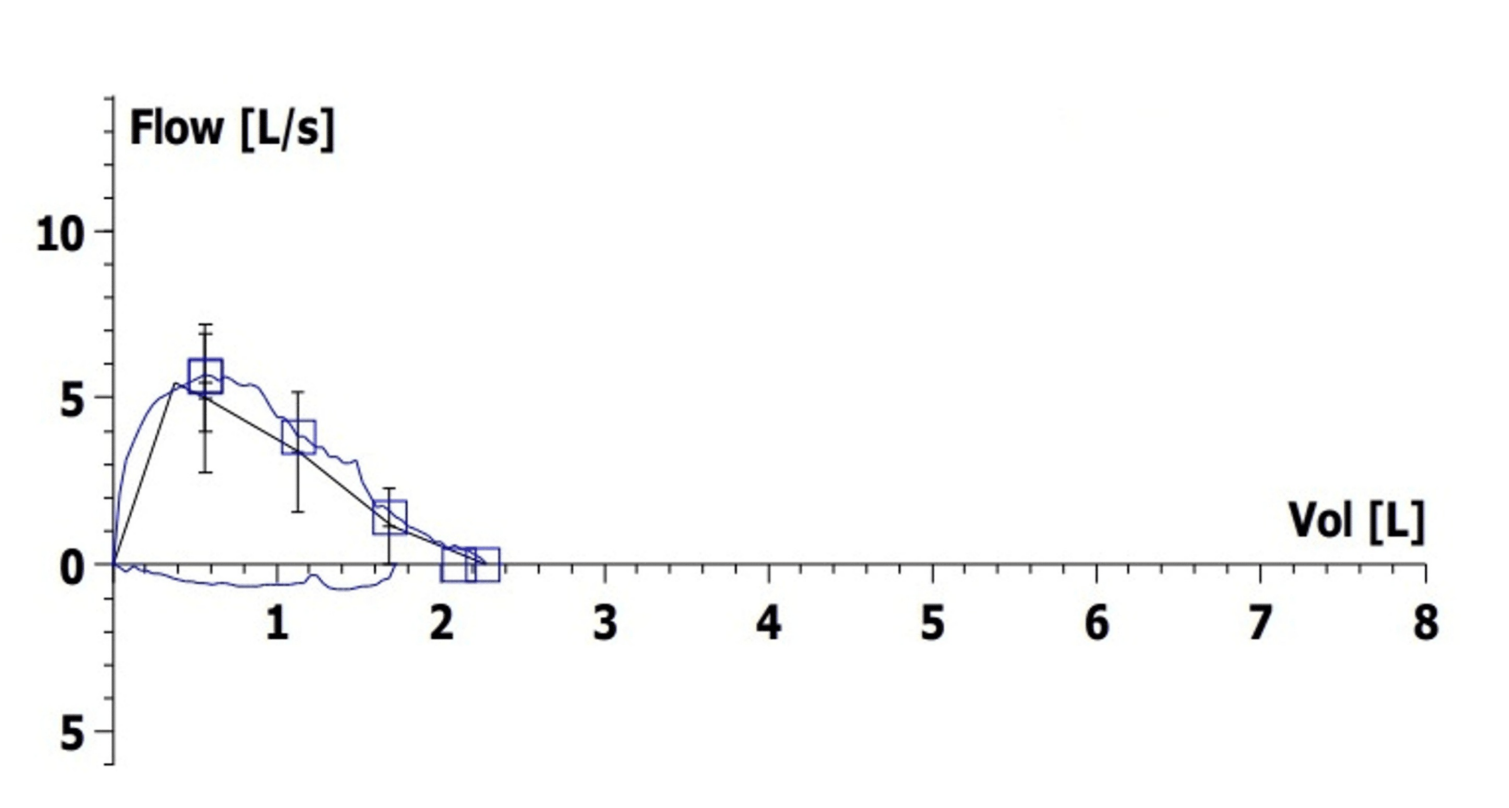
Spirometry flow-volume loop of the patient at age 64 years.

Riboflavin supplementation (10 mg/kg/day) was initiated at age 63 but discontinued after three years due to lack of improvement. Slowly worsening inspiratory stridor was documented after phonation pauses. Laryngofibroscopy revealed vocal cords in adduction, slightly moving during phonation, and reduced glottic cleft. She declined cochlear implants and started language therapy. Other than stridor, she reported no respiratory symptoms (dyspnea, poor sleep quality, morning headaches, or daytime sleepiness). Initial respiratory function tests displayed normal forced vital capacity (FVC) (2.36 L, 101.5% predicted) with both reduced maximal inspiratory pressure (MIP) and maximal expiratory pressure (45.4 and 48.9 cmH_2_O, respectively). Spirometry flattened inspiratory flow-volume loop suggested a variable extrathoracic obstruction (Figure 1), explained by vocal cord paresis. Arterial blood gas analysis and nocturnal oximetry were normal.

In a year, MIP significantly declined (20.70 cmH2O), while FVC remained stable. Therefore, nocturnal non-invasive mechanical ventilation (NIV) Bilevel-ST was initiated, but temporarily suspended due to pressure intolerance. Peak cough flow (PCF) was 210 L/minute. Although there were no respiratory infections and rare choking episodes, regular air-stacking manoeuvres were initiated (15 repetitions three times a day), reserving mechanical insufflation-exsufflation on-demand access. Further evaluation immediately after air stacking documented a PCF of 312 L/minute. With stable BMI (19.0 kg/m²), percutaneous endoscopic gastrostomy was deferred in favour of regular swallowing monitoring and use of thickened pureed diet. Discussions on tracheostomy are ongoing. An overview of the patient's clinical timeline is presented in Table 1.

**Table 1:** Patient clinical timeline overview, since birth to current status NIV: non-invasive ventilation; S/T: Spontaneous/Timed; PEG: Percutaneous Endoscopic Gastrostomy

Age (years)	Clinical Event / Finding
0-11	Normal birth and early development
12	Onset of mild hearing loss
Teens–Adulthood	Progressive sensorineural deafness and bilateral facial weakness; bilateral paresis of VII, IX, X, and XII cranial nerves
62	Profound deafness; bilateral facial palsy, tongue atrophy/fasciculations, dysphonia, dysphagia; brisk deep tendon reflexes in the limbs
63	Riboflavin supplementation started (10 mg/kg/day); discontinued after 3 years due to lack of response
64–65	Emergence of inspiratory stridor; respiratory workup initiated
65	Nocturnal NIV (Bilevel-S/T mode) started, although temporarily suspended due to intolerance
68	Regular air-stacking regimen initiated (15 reps, 3x/day); mechanical insufflation-exsufflation available on demand; nocturnal NIV regular use; thickened pureed diet, PEG deferred; tracheostomy under discussion

## Discussion

Clinical descriptions of BVVLS are limited due to its rare prevalence. This case presents a unique phenotype that aligns broadly with BVVLS but also presents atypical features and some diagnostic uncertainty, highlighting the variability in presentation and the challenges in establishing a definitive diagnosis. Typical BVVLS characteristics included childhood onset, initial neurosensory deafness, progressive cranial nerve involvement, preserved intellect, and normal brain MRI [2,3,14]. In fact, *SLC52A3* disease often has a later onset (10-30 years of age), more respiratory involvement, and facial weakness than *SLC52A2* mutations [14,18,19]. Our patient had symptom onset at 12 years of age, progressive facial weakness, and required NIV plus assisted clearance airway techniques due to respiratory involvement. This is coherent with the *SLC52A3* disease, but in the mildest phenotype spectrum. The most notable aspect of this case is the unusually slow progressive clinical course with significantly longer survival, receiving only brief riboflavin supplementation at 63 years. In contrast, median survival for untreated BVVLS is described as 7.5 years from symptom onset [18].

Currently at 68 years, the patient in this report is the second-oldest case documented, after a 71-year-old man was reported in 2022 [18], who consumed riboflavin-rich foods; no such dietary habit was observed here. A possible explanation for our patient exhibiting a milder phenotype, slow progression over decades, and longer survival may lie in the fact that a single heterozygous *SLC52A3* gene variant (c.1238T>C) was identified. It was classified by our reference laboratory as a variant of unknown significance because it has only been described in compound heterozygous patients [15-17]. Traditionally, BVVLS follows autosomal recessive inheritance, but more recently, heterozygous *SLC52A3* variants have been reported in symptomatic RTD patients with longer survival rates [20,21]. In fact, it is hypothesized that in individuals harboring only a single mutation in the *SLC52A2* or *SLC52A3* gene, such as in our patient, a second pathogenic alteration may still exist on the other allele but remains undetected [11]. This undetected variant could involve a heterozygous copy number variant or a mutation in non-coding regions of the *SLC52* gene, including deep intronic or promoter variants [11]. These limitations clearly underscore the need for further research into the genetic landscape of BVVLS.

Currently, high-dose riboflavin supplementation (10-80 mg/kg/day) remains the only lifesaving treatment, but presents variable clinical improvement: 50% strong and 36% mild responses [3,19,21]. Although also variable, treatment responses have been reported within the first few days to a year of therapy [12]. However, late treatment, as in this case, often yields no improvement, reflecting established neuronal damage [14,22]. 

A multidisciplinary approach is required for supportive management [23]. Respiratory failure, due to muscle weakness and diaphragmatic paralysis, is the leading cause of BVVLS mortality [14]. Bosch et al. described respiratory symptoms in 64%, stridor in 25% and diaphragm weakness/palsy in 15% of BVVLS [12]. Most patients with respiratory failure require mechanical ventilatory support with NIV or tracheostomy, even during childhood [2,14]. Although riboflavin supplementation can improve respiratory symptoms and diaphragm function, even leading to NIV withdrawal or tracheostomy closure, respiratory failure remains the main cause of death [2,12,14]. Ventilatory support experience for BVVLS is limited and primarily extrapolated from neuromuscular diseases with similar bulbar involvement, such as amyotrophic lateral sclerosis (ALS). In ALS, NIV is typically indicated when FVC falls below 50% of predicted (but strongly considered when <70%) or MIP is less than 60 cmH₂O [24]. Accordingly, in this case, nocturnal NIV was initiated not only because MIP remained persistently below 60 cmH₂O, but also due to its marked decline over initial follow-up. In this context, pulmonology plays a crucial role in the clinical management of these patients, particularly in monitoring respiratory function, initiating NIV early, and implementing airway clearance techniques.

This case report aims to raise awareness of this rare diagnostic entity, with potential for underdiagnosis in cases with milder phenotypes. It also emphasizes the current challenges in diagnosing similar cases, while advocating for further research into the genetic transmission patterns and epigenetics of this still poorly understood condition.

## Conclusions

This case highlights the possibility of a rare diagnostic entity, BVVLS, presenting with a clinically compatible phenotype. However, the unique presentation, characterized by prolonged survival and the slow, mild progression of symptoms, possibly linked to a single heterozygous *SLC52A3* variant, does not fully align with the classic autosomal recessive inheritance pattern or the rapid deterioration typically observed in BVVLS cases. This raises the possibility of an atypical form of the disease, for which the authors aim to raise awareness, while emphasizing the need for further research into the genetic transmission patterns and epigenetics of this still poorly understood condition.

While riboflavin supplementation remains the cornerstone therapy for RTD, this case underscores its limited effectiveness when initiated at an advanced stage. These findings highlight the importance of early genetic diagnosis and the timely initiation of treatment, while also illustrating the variability in the phenotypic spectrum of BVVLS. Additionally, this case underscores the critical role of pulmonology in the multidisciplinary management of BVVLS. The integration of nocturnal NIV and advanced airway clearance techniques, adapted from the management of other neuromuscular diseases, proved essential in addressing the patient's progressive respiratory compromise. Respiratory interventions significantly improved the patient’s quality of life and may have prolonged survival. We reinforce the need for continued research into genetic and therapeutic determinants of BVVLS, while also demonstrating how tailored multidisciplinary care can mitigate the impact of rare diseases with severe systemic involvement.
